# Rapidly generating knockout mice from *H19-Igf2* engineered androgenetic haploid embryonic stem cells

**DOI:** 10.1038/celldisc.2015.31

**Published:** 2015-11-03

**Authors:** Meili Zhang, Yufang Liu, Guang Liu, Xin Li, Yuyan Jia, Lihong Sun, Liu Wang, Qi Zhou, Yue Huang

**Affiliations:** 1 State Key Laboratory of Medical Molecular Biology, Institute of Basic Medical Sciences, Chinese Academy of Medical Sciences & Peking Union Medical College, Beijing 100005, China; 2 Department of Medical Genetics, Institute of Basic Medical Sciences, Chinese Academy of Medical Sciences & Peking Union Medical College, Beijing 100005, China; 3 State Key Laboratory of Reproductive Biology, Institute of Zoology, Chinese Academy of Sciences, Beijing 100101, China; 4 Center for Experimental Animal Research, Institute of Basic Medical Sciences, Chinese Academy of Medical Sciences & Peking Union Medical College, Beijing 100005, China

**Keywords:** Haploid ES cells, AG-haESCs, imprinted gene, ICAHCI, gene-modified mice

## Abstract

Haploid mammalian embryonic stem cells (ESCs) hold great promise for functional genetic studies and assisted reproduction. Recently, rodent androgenetic haploid ESCs (AG-haESCs) were generated from androgenetic blastocysts and functioned like sperm to produce viable offspring via the intracytoplasmic AG-haESCs injection into oocytes. However, the efficiency of this reproduction was very low. Most pups were growth-retarded and died shortly after birth, which is not practical for producing knockout animals. Further investigation suggested a possible link between the low birthrate and aberrant expression of imprinted genes. Here, we report the high-frequency generation of healthy, fertile mice from *H19-Igf2* imprinting-locus modified AG-haESCs, which maintained normal paternal imprinting and pluripotency. Moreover, it is feasible to perform further genetic manipulations in these AG-haESCs. Our study provides a reliable and efficient tool to rapidly produce gene-modified mouse models and will benefit reproductive medicine in the future.

## Introduction

Recently, mammalian haploid embryonic stem cell (ESC) lines were successfully established in mouse, rat and monkey [1–4]. They displayed unique potential for functional genetic studies and hold great promise for assisted reproduction [5, 6]. Moreover, androgenetic haploid ESCs (AG-haESCs) were isolated from androgenetic rodent blastocysts and functioned like sperm to produce viable, fertile progeny after intracytoplasmic injection into mature oocytes [3, 7, 8], providing a new approach for rapidly producing genetically modified mice. However, the efficiency was extremely low, and many of these semi-cloned (SC) pups were growth-retarded and died shortly after birth, most likely due to instable paternal imprinting in these AG-haESCs.

Genomic imprinting has an essential role in mammalian development [9–11]. Among the ~150 reported murine imprinted genes, the *H19-Igf2* locus was first identified and shown to be essential for normal fetal growth [12, 13]. Furthermore, viable bi-maternal mice were produced from reconstructed eggs containing fully grown oocytes and non-growing oocytes that harbored a deletion in the *H19-Igf2* locus [14]. Interestingly, abnormal *H19* imprinting was observed in the prolonged cultured AG-haESCs and growth-retarded newborns mentioned above [7, 8]. We therefore asked whether genetic modification of the *H19-Igf2* locus in AG-haESCs could yield fertile transgenic mice at a high frequency.

## Results

### Genetic modification of the H19-Igf2 locus in AG-haESCs

**Figure 1:**
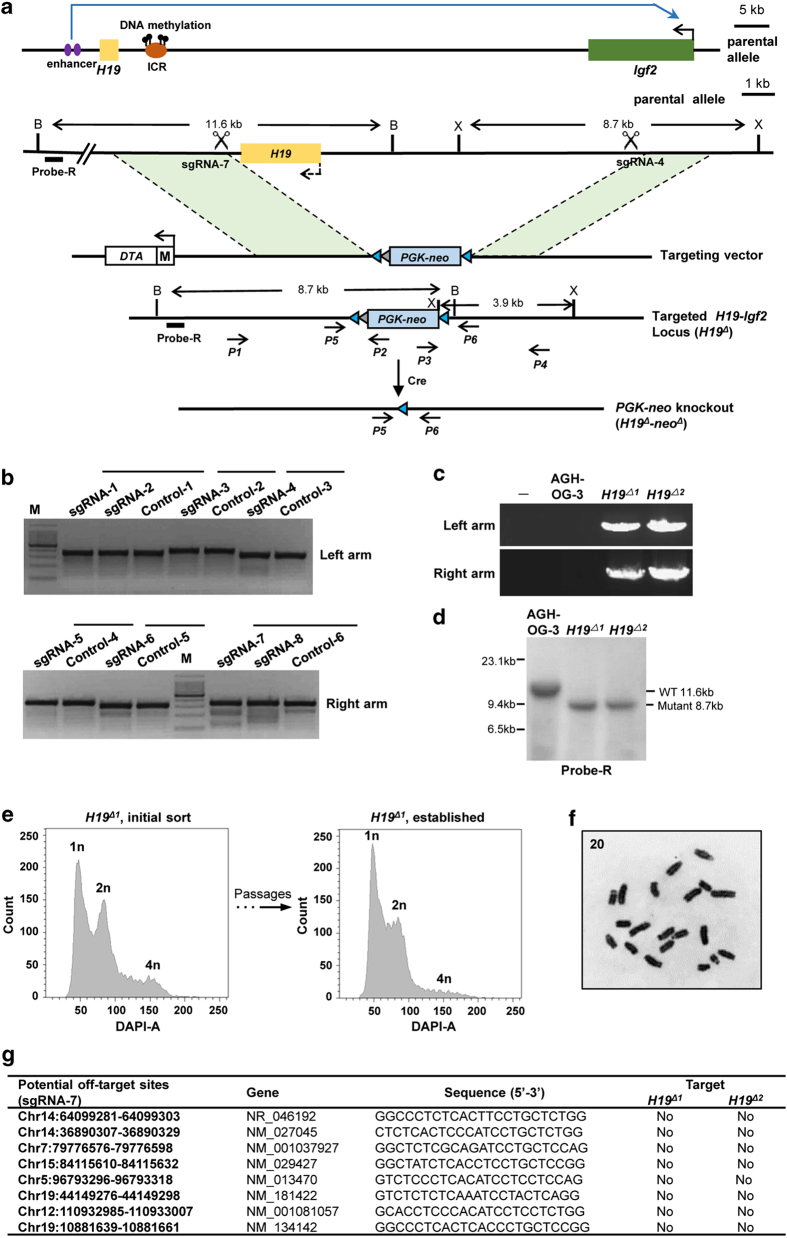
Genetic modification of the *H19-Igf2* locus in AG-haESCs. (**a**) Schematic representation of CRISPR-Cas9 assisted homologous recombination to target the *H19-Igf2* locus in AG-haESCs (*H19*^*Δ*^AG-haESCs). The *PGK-neo* cassette was deleted by Cre (*H19*^*Δ*^*-neo*^*Δ*^AG-haESCs). (**b**) Surveyor assay for Cas9-mediated cleavage up- and downstream of the *H19* locus in AG-haESCs. (**c**) Validation of gene targeting in *H19*^*Δ*^AG-haESCs by PCR. (**d**) Confirmation of gene targeting in *H19*^*Δ*^AG-haESCs by Southern blot. (**e**) Establishment of the *H19*^*Δ1*^AG-haESC line after FACS enrichment for haploid cells. (**f**) Karyotype of *H19*^*Δ1*^ AG-haESCs showing normal haploidy. (**g**) Identification of the potential off-targets of CRISPR-Cas9 in *H19*^*Δ*^AG-haESCs.

The mouse *H19* gene produces a 2.3 kb long non-coding RNA exclusively expressed from the maternal allele and physically linked to the *Igf2* gene on chromosome 7. They are reciprocally imprinted. The imprinting control region (ICR) within the *H19-Igf2* locus is essential for transcriptional insulation of the maternal *Igf2* allele [15]. To disrupt *H19* gene expression in AG-haESCs, we used clustered regularly interspaced short palindromic repeat (CRISPR)-Cas9 enhanced homologous recombination [16, 17] to knock out the 13 kb region of *H19* that includes the transcription unit and the ICR (Figure 1a). We designed four single guide RNAs (sgRNAs) targeting different sites up- or downstream of the region and investigated the specificity of these sgRNAs via the Surveyor assay [18]. Cas9/sgRNA-4 and Cas9/sgRNA-7 transfection efficiently cleaved at the target loci and were used for gene targeting (Figure 1b). We co-transfected the pCCI-*H19*, Cas9-sgRNA-4 and Cas9-sgRNA-7 plasmids into AGH-OG-3 AG-haESCs harboring the *Oct4* promoter-driven *eGFP* (*Oct4-eGFP*) transgene. The positive clones were examined by PCR (Figure 1c) and were further validated by Southern blot analysis (Figure 1d). Among the 96 picked colonies, 29 positive clones harbored the desired *H19-ICR* deletion (*H19* ^*Δ*^). We randomly chose two clones containing considerable haploid cells for further experiments. The two AG-haESC lines, referred to as *H19*^*Δ1*^ and *H19*^*Δ2*^, were established through consecutive passages and multiple rounds of fluorescence-activated cell sorting (FACS) for haploid cells (Figure 1e). Karyotyping analysis showed that these cells contained a haploid set of 20 chromosomes (Figure 1f). Comparative genomic hybridization (CGH) analysis confirmed the genome integrity of the two haploid cell lines (Supplementary Table S1). Analyses of potential off-target regions showed that none of the eight predicted sites were mutated by CRISPR/Cas9 in the two-cell clones (Figure 1g).

### Characteristics of H19^Δ^ AG-haESCs

**Figure 2:**
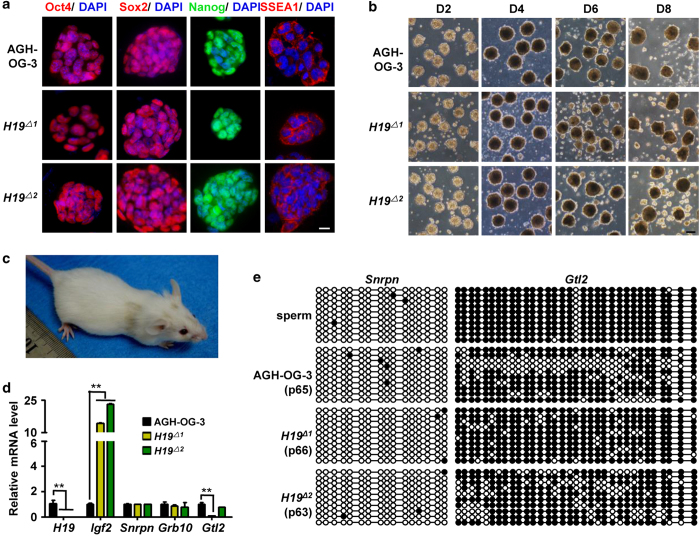
Characteristics of *H19*^*Δ*^AG-haESCs. (**a**) Immunofluorescence staining of AGH-OG-3 and *H19*^*Δ*^AG-haESCs. Scale bar, 20 μM. (**b**) Different days of embryoid bodies (EB) formation of AGH-OG-3 and *H19*^*Δ*^AG-haESCs. Scale bar, 200 μM. (**c**) Adult chimeric mouse produced by microinjection of haploid *H19*^*Δ1*^AG-haESC into diploid blastocysts. (**d**) Expression of imprinted genes measured by quantitative reverse transcription PCR (RT-qPCR). AGH-OG-3 AG-haESCs were used as control. Error bars, ±s.d. *n*=3. **P<0.01. (**e**) Methylation analysis of the *Snrpn* and *Gtl2* DMRs in *H19*^*Δ*^AG-haESCs. Sperm DNA was used as control. Open circles represent unmethylated CpG sites, whereas filled circles represent methylated CpG sites.

Cultured *H19*^*Δ*^ AG-haESCs exhibited dome-shaped colony morphology similar to diploid mouse ESCs and expressed the pluripotency genes *Oct4*, *Sox2*, *Nanog* and *SSEA1* (Figure 2a). These cells formed embryoid bodies when cultured in suspension (Figure 2b). The pluripotency was further tested by injection of the *H19*^*Δ*^AG-haESCs into diploid ICR blastocysts derived from mice with white coats. One chimeric mouse was obtained and survived to adulthood without obvious defects (Figure 2c). These studies provided evidence for the pluripotency of *H19*^*Δ*^ AG-haESCs.

We then analyzed whether the *H19-ICR* deletion affected the imprinting status of AG-haESCs. We first examined the expression of *H19*, *Igf2*, *Snrpn* (maternally imprinted), *Grb10* (paternally imprinted) and *Gtl2* (paternally imprinted) in AGH-OG-3 and the two *H19*^*Δ*^haploid cell lines. As expected, the expression of *H19* was nearly undetectable in the two *H19*^*Δ*^ cell lines, whereas the *Igf2* level was significantly upregulated (Figure 2d). *Snrpn* and *Grb10* expression in *H19*^*Δ*^AG-haESCs was comparable to expression in AGH-OG-3 AG-haESCs (Figure 2d). Interestingly, the expression level of *Gtl2* in the two *H19*^*Δ*^ cell lines was varied. *Gtl2* was expressed at low levels in *H19*^*Δ1*^AG-haESCs, whereas its expression in *H19*^*Δ2*^AG-haESCs was similar to AGH-OG-3 cells (Figure 2d). This demonstrated the intraclonal heterogeneity of *Gtl2* expression in AGH-OG-3 cells. To further assess the DNA methylation profile of the imprinted genes, we performed bisulfite sequencing to analyze the ICRs of the *Snrpn* and *Gtl2* loci. *H19*^*Δ1*^AG-haESCs showed normal methylation patterns similar to sperm in the ICRs of *Snrpn* and *Gtl2*, whereas late-passage (p65) AGH-OG-3 cells displayed abnormal methylation in the *Gtl2* ICR. *H19*^*Δ2*^AG-haESCs harbored an abnormal methylation of the *Gtl2* ICR similar to AGH-OG-3 cells (Figure 2e). The normal *Gtl2* ICR methylation in *H19*^*Δ1*^AG-haESCs indicated that some late-passage AGH-OG-3 cells maintained normal imprinting of the *Gtl2* ICR. The methylation status of the *Snrpn* and *Gtl2* ICRs in *H19*^*Δ1*^ *and H19*^*Δ2*^AG-haESCs was consistent with the expression of *Snrpn* and *Gtl2* in these cells. These results indicated that *H19-ICR* deletion in AG-haESCs had a negligible impact on the imprinting status of genes except for the *H19-Igf2* locus.

### H19^Δ^ AG-haESCs efficiently support the generation of healthy SC mice

**Figure 3:**
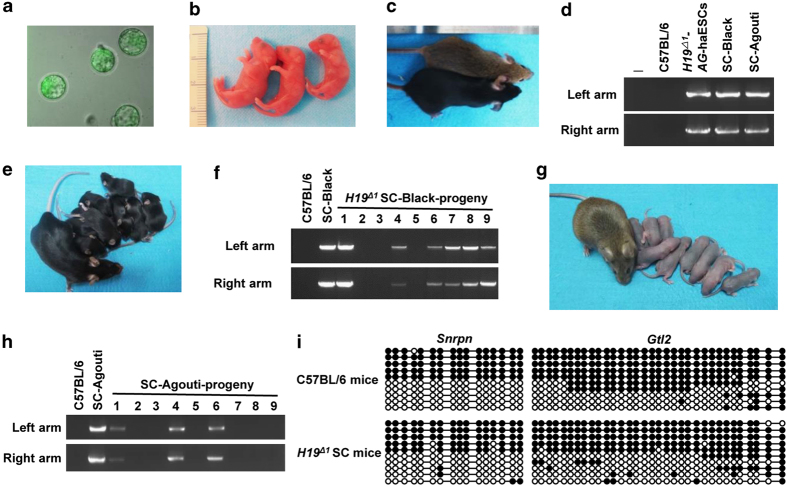
Generation of ICAHCI offspring by *H19*^*Δ1*^AG-haESCs. (**a**) Blastocysts generated by injection of *H19*^*Δ1*^AG-haESCs into oocytes. The *H19*^*Δ1*^AG-haESCs carried the *Oct4-eGFP* transgene. (**b**) Three SC pups from ICAHCI using *H19*^*Δ1*^AG-haESCs. (**c**) Adult SC mice derived from ICAHCI using *H19*^*Δ1*^AG-haESCs. (**d**) PCR analysis of *H19* deletion in SC mice. (**e**) Adult *H19*^*Δ1*^SC-Black mouse and its progeny. (**f**) PCR analysis of *H19* deletion in the progeny of *H19*^*Δ1*^SC-Black mice. The primer pairs P1–P2 and P3–P4 were used. (**g**) Adult *H19*^*Δ1*^SC-Agouti mouse and its progeny. (**h**) PCR analysis of the *H19* deletion in the progeny of SC-Agouti mice. (**i**) Methylation analysis of imprinting in *H19*^*Δ1*^SC pups. C57BL/6 mice DNA was used as control. Open circles represent unmethylated CpG sites, whereas filled circles represent methylated CpG sites.

We were interested in testing whether *H19-ICR* deletion in AG-haESCs increased the efficiency of producing offspring. We injected FACS-sorted G0- or G1- phase *H19*^*Δ*^AG-haESCs into pre-activated oocytes via intracytoplasmic AG-haESCs injection (ICAHCI). *H19*^*Δ*^AG-haESCs contributed to embryos, as judged by *Oct4-eGFP* expression in developed blastocysts (Figure 3a). We transferred 80 two-cell embryos from the *H19*^*Δ1*^AG-haESC line into two pseudopregnant ICR mice and obtained three female full-term pups. All pups had normal body size and did not exhibit obvious developmental retardation after birth (Figure 3b). Two pups grew to adulthood with black (SC-Black) and agouti (SC-Agouti) fur, respectively (Figure 3c and d). Because *H19*^*Δ1*^AG-haESCs had a C57BL/6 background, the black or agouti coat colors of SC mice depended on the oocytes used for ICAHCI, which were derived from CD-1 or B6D2F1 (C57BL/6×DBA/2) mice, respectively. The SC mice delivered healthy progeny with litter sizes of 8–10 when mated with C57BL/6 males (Figure 3e and g). We obtained three SC-Black litters and two SC-Agouti litters. Approximately half of these carried the *H19-ICR* deletion, consistent with the expected Mendelian ratio (Figure 3f and h). The rate of SC mice born was ~4% of *H19*^*Δ1*^AG-haESCs ICAHCI (Table 1) compared with the ~2.2% efficiency of generating SC mice from early passage AGH-OG-3 (passage 17) AG-haESCs ICAHCI and 0% from late-passage AGH-OG-3 cells (passage 22) [7]. Many of the AGH-OG-3 AG-haESC derived pups were growth-retarded and died within 1 h of birth [7]. However, no growth-retarded pups were produced from *H19*^*Δ1*^AG-haESCs ICAHCI, even though the cells were from a very late passage (more than passage 56). The growth retardation of SC mice might be due to the loss of methylation in the *H19* ICR and the consequent lower expression of *Igf2*. In SC mice generated by *H19*^*Δ1*^AG-haESCs ICAHCI, *H19-ICR* was deleted, leading to the normal expression of *Igf2*. The methylation status of two other imprinted genes, *Snrpn* and *Gtl2,* was normal in SC mice (Figure 3i). These results demonstrated that *H19*^*Δ*^AG-haESCs could function like sperm to produce normal mice and transmit their genetic material to progeny. Thus, modification of the *H19-Igf2* locus was sufficient to generate healthy and fertile SC mice at a high efficiency.

### Generation of genetically modified mice using H19^Δ^ AG-haESCs

**Figure 4:**
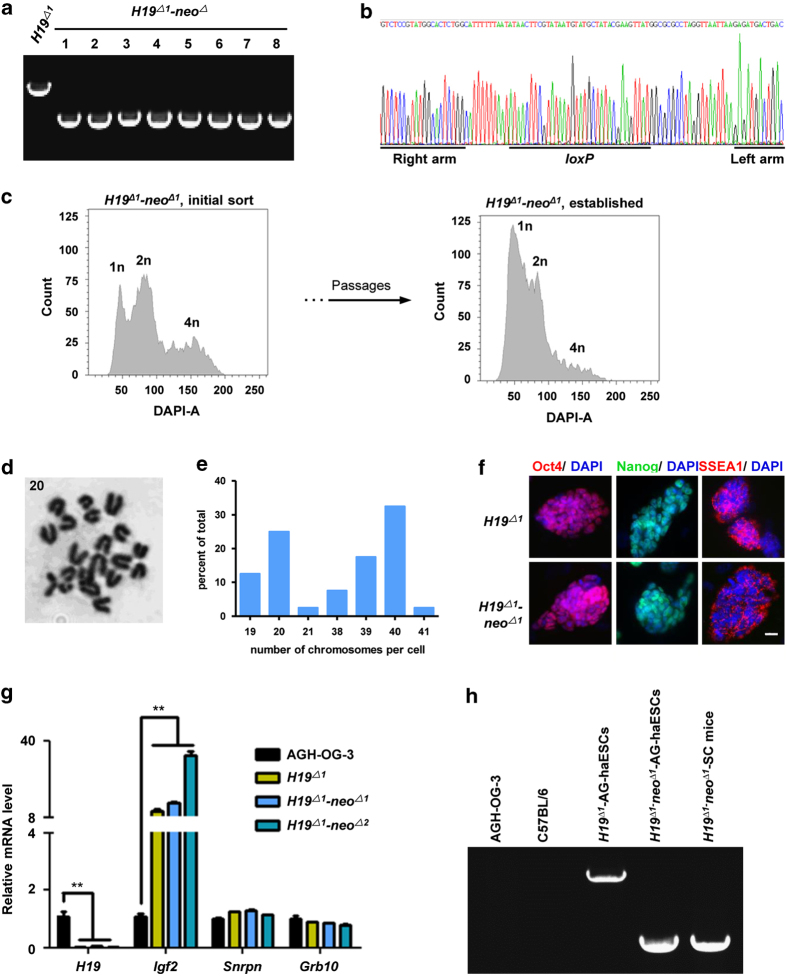
Generation of ICAHCI offspring by *H19*^*Δ1*^*-neo*^*Δ*^AG-haESCs. (**a**) Subclones of *PGK-neo* knockouts in *H19*^*Δ1*^AG-haESCs by PCR. The primer pairs P5–P6 were used. (**b**) DNA sequence of PCR products amplified from the *H19* gene of *H19*^*Δ1*^*-neo*^*Δ*^AG-haESCs. (**c**) Establishment of the *H19*^*Δ1*^*-neo*^*Δ1*^AG-haESC line after FACS enrichment for haploid cells. (**d**) Chromosome counting in *H19*^*Δ1*^*-neo*^*Δ1*^AG-haESCs showing normal haploidy. (**e**) The distribution of absolute chromosome numbers of *H19*^*Δ1*^*-neo*^*Δ1*^AG-haESCs under cell culture conditions. (**f**) Immunofluorescence staining of *H19*^*Δ*^and *H19*^*Δ1*^*-neo*^*Δ1*^AG-haESCs. Scale bar, 20 μM. (**g**) Expression of imprinted genes measured by quantitative reverse transcription PCR (RT-qPCR). AGH-OG-3 AG-haESCs were used as control. Error bars, ±s.d. *n*=3. **P<0.01. (**h**) PCR analysis of *PGK-neo* knockout in *H19*^*Δ1*^*-neo*^*Δ1*^SC mice.

To further analyze the feasibility of producing genetically modified mouse models from *H19*^*Δ*^AG-haESCs, we deleted a *loxP* flanked *PGK-neo* cassette by transient expression of Cre in the *H19*^*Δ1*^AG-haESC line (Figure 1a). Among the 96 picked clones, the *PGK-neo* cassette was correctly deleted in most clones, as analyzed by PCR (Figure 4a). The remaining *loxP* site in the *H19-Igf2* locus was further confirmed by DNA sequencing (Figure 4b). We then randomly chose 24 correctly deleted clones (*H19*^*Δ1*^*-neo*^*Δ*^) and performed FACS to determine the haploid subpopulation in these clones. The haploid ESC lines were established through consecutive passages, followed by two rounds of FACS for haploid cells (Figure 4c). The *H19*^*Δ1*^*-neo*^*Δ*^ AG-haESCs had normal haploid karyotypes (Figure 4d and e), expressed pluripotency markers (Figure 4f), and imprinted genes comparable to *H19*^*Δ*^AG-haESCs (Figure 4g). After injecting the sorted G0- or G1- phase *H19*^*Δ1*^*-neo*^*Δ1*^AG-haESCs into the pre-activated oocytes, we successfully obtained 50 two-cell stage embryos and transplanted them into pseudopregnant females. One full-term live pup derived from the *H19*^*Δ1*^*-neo*^*Δ1*^AG-haESC line was obtained (Table 1). Genotype analysis confirmed *PGK-neo* cassette deletion in the SC animal (Figure 4h). Our results demonstrated that *H19*^*Δ*^AG-haESCs can be used to rapidly generate gene-modified mice.

## Discussion

Although we do not know why the paternally imprinted gene *H19* easily lost methylation in AG-haESCs, successful restoration of *Igf2* expression by *H19-ICR* deletion faithfully improved the efficiency to generate viable SC mice. Other imprinted genes, such as *Grb10*, *Dlk1* and *Gtl2*, also affect embryonic growth [11]. Moreover, *Dlk1-Dio3* ICR deletion significantly increased the efficiency of generating parthenogenetic mice [14, 19]. It is tempting to investigate whether additional modification of these imprinted genes in AG-haESCs would increase the efficiency of producing SC mice, although the expression level and methylation status of these genes in AG-haESCs were comparable to those in sperm [7, 8]. During the submission of this work, an independent study [20] reported the highly efficient generation of healthy and fertile SC pups from AG-haESCs carrying deletions in the *H19*-DMR alone or in addition with *Gtl2*-DMR, confirming our findings. *Gtl2*-DMR deletion ensured the full silencing of the paternally imprinted gene *Gtl2* in AG-haESCs, consistent with the normal imprinting status of *Gtl2* in *H19*^*Δ1*^AG-haESCs.

Modification of the *H19-Igf2* locus in AG-haESCs faithfully improved the efficiency of generating healthy SC mice via the ICAHCI technology. Our work provides another approach for quickly generating gene-modified mice in addition to the VelociMouse [21] and CRISPR-Cas9 [22] technologies. The *H19*^*Δ*^AG-haESCs have ‘sperm-like’ activity and can replace sperm in reproduction to overcome their limitations. Sperm are not able to propagate and are thus difficult to genetically modify *in vitro*. In addition, many established methods and resources for genetic studies of diploid ES cells are immediately applicable to *H19*^*Δ*^AG-haESCs. This work provides inspiration for possible human AG-haESC research and may benefit male infertility in the future, similar to the contribution of spermatogonial stem cells [23].

## Materials and methods

### Mice

All mouse experimental protocols were approved by the Institutional Animal Care and Use Committee at Peking Union Medical College & Chinese Academy of Medical Sciences. And all animal care and experimental methods were carried out in accordance with the institutional ethical guidelines for animal experiments. Male mice of C57BL/6 were used for sperm collection. B6D2F1 (C57BL/6×DBA/2) and CD-1 female mice were used to provide oocytes for micromanipulation. Female mice of ICR were used to provide blastocysts for microinjection and were also used as pseudopregnant mice. C57BL/6 mice were used for animal mating.

### AG-haESCs

Androgenetic haploid ES cell line AGH-OG-3 was obtained from Dr Jinsong Li’s lab in Shanghai (China). The cells were cultured in ESC medium supplemented with 15% fetal bovine serum, 1 000 U ml^−1^ leukemia inhibitory factor, 3  μM CHIR99021 and 1 μM PD0325901.

### DNA content analysis

The dissociated cells were incubated with 10 μg ml^−1^ Hoechst 33342 at 37 °C for 30 min. Then the haploid 1n peak was purified by flow-cytometry (BD FACSAria III, San Jose, CA, USA). Flow-cytometric data were analyzed using the BD FACSDiva software.

### Vector construction

For pCCI-*H19* construction, the left and right homologous arms were amplified from bacterial artificial chromosome (RP23-209022). *Eag* I and *Pac* I restriction sites were added to the amplified left homologous arm, and *Sma* I and *Xho* I sites were added to the right arm. *Eag* I and *Pac* I digested left arm was cloned into pCCI18 backbone (digested with *Not* I and *Pac* I). The products were then digested with *Xho* **I** and ligated with *Sma* I and *Xho* I digested right arm.

### Construction of CRISPR plasmids

The pX330 plasmid was bought from Addgene, Cambridge, MA, USA. The sgRNA-1, -2, -3, -4, -5, -6, -7 and -8 oligos were synthesized, annealed and ligated to the pX330 plasmid that was digested with *B*bs I (New England Biolabs, Ipswitch, MA, USA). The sequences of designed sgRNAs were listed in Supplementary Table S2.

### Surveyor assay

The AG-haESCs were transfected with CRISPR-sgRNA plus pPB-puro plasmids and selected by puromycin for 36 h. Genomic DNA from wild-type and transfected AG-haESCs was extracted. PCR products were denatured, annealed and treated with T7EN1 (New England Biolabs). The primers used for PCR were listed in Supplementary Table S2.


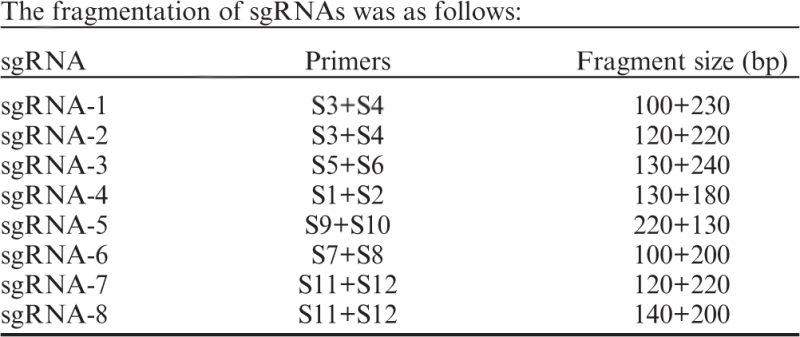


### Gene targeting in AGH-OG-3 AG-haESCs

The pCCI-*H19*, Cas9-sgRNA-4 and Cas9-sgRNA-7 plasmids were co-transfected into AGH-OG-3 AG-haESCs. The cells were selected by G418 (150 μg/ml) for 7 days. Colonies were picked and analyzed by PCR and flow cytometer sorting. The CAGGS-Cre plasmid was electroporated into *H19*^*Δ1*^AG-haESCs to delete the *loxP* flanked *PGK-neo* cassette.

### Southern blotting

Wild-type and *H19*^*Δ*^AG-haESC genomic DNA was digested using *Bgl*II restriction enzyme. The digested DNA was subsequently separated on a 1% agarose gel for 5 h, and then transferred to a nylon membrane and hybridized with α-^32^P random primer-labeled probes.

### Prediction of potential off-targets

The potential off-target regions were predicted using the online tool, http://crispr.mit.edu/. The regions with score >0.5 and mismatch ⩽4 were considered as the potential off-target sites. They were amplified using High-Fidelity DNA Polymerase (New England Biolabs) and sequenced.

### Intracytoplasmic injection

ICAHCI was described as previously [8]. In brief, mature oocytes were collected from the oviduct of super-ovulated female B6D2F1 and CD-1 mice and were pre-activated by 10 mM SrCl_2_ in calcium-free CZB medium for 30 min before microinjection. G0- or G1-phase purified AG-haESCs were collected and injected into oocytes separately. When the constructed embryos developed to the 2-cell stage in KSOM-AA medium (Sigma, St Louis, MO, USA), they were transferred to the oviduct of pseudopregnant ICR mice at 0.5 d.p.c.

### Comparative genomic hybridization analysis

The genomic DNA of wild-type and *H19*^*Δ*^ AG-haESCs was extracted using the Wizard Genomic DNA Purification Kit (Promega, Madison, WI, USA) and sent to the CapitalBio Corporation (Changping District, Beijing, China) for CGH analysis. The SurePrint G3 Mouse CGH 4×180 K microarrays (Agilent, Santa Clara, CA, USA) were used.

### Karyotype analysis

Exponentially growing ES cells were incubated with 0.2 μg ml^−1^ colcemid (Sigma) for 2–3 h at 37 °C. After trypsinization, the collected cells were incubated in 0.075 M KCl hypotonic solution for 15 min at 37 °C. Hypotonic solution-treated ES cells were fixed in fresh ice cold 3:1 methanol/acetic acid at room temperature and dropped onto the precleaned slides. The chromosome spreads were stained with Giemsa solution (10% v/v Giemsa to 7.0 pH phosphate buffer) for 5 min and washed in ddH_2_O. More than 30 metaphase spreads were analyzed.

### Immunostaining

ES cells on coverslips were fixed in 4% paraformaldehyde/ phosphate buffer saline (PBS) for 15 min at room temperature. The fixed cells were then permeabilized with 0.5% Triton X-100 for 10 min and were blocked in 3% bovine serum albumin for 1 h at room temperature. The cells were incubated overnight at 4 °C with primary antibodies, anti-oct3/4 (sc-5279; Santa Cruz, Dallas, TX, USA), anti-sox2 (sc-17320; Santa Cruz), anti-nanog (AB5731; Millipore, Billerica, MA, USA), and anti-SSEA1 (sc-21702; Santa Cruz). The cells were treated with the secondary antibodies for 1 h at room temperature. The nuclei were dyed with 4′,6-diamidino-2-phenylindole (DAPI). Images were acquired by the fluorescent microscope (Zeiss, Oberkochen, Germany).

### Quantitative reverse transcription PCR

Total RNA was extracted from AG-haESCs using Trizol reagent (Invitrogen, Waltham, MA, USA). 1.0 μg of total RNA was reverse transcribed using the PrimeScript II 1st strand cDNA synthesis kit (TaKaRa, Dalian, China). The real-time PCR reaction was performed using SYBR Premix Ex Taq II (TaKaRa) and run on Roche 480 Light Cycler. The amount of GAPDH expression was used to normalize all values.

### Bisulphite sequencing

Genomic DNA of ESCs, sperm and mouse tail was treated with the EpiTect Bisulphite (Qiagen, Hilden, Germany) for bisulphite conversion according to the manufacturer’s instructions. Differentially methylated regions (DMRs) of *Snrpn* and *Gtl2* were amplified. The PCR products were cloned into pMD18-T vectors (TakaRa) and sequenced for unmethylated C to T conversion. Bisulphite primers are presented in Supplementary Table S2.

### Embryoid-body formation

Embryoid bodies were formed from wild-type and *H19*^*Δ*^AG-haESCs. Cultured ES cells were dissociated with trypsin and sedimentated for 30 min at 37 °C. 1.5×10^6^ cells were transferred to low attachment 90-mm-diameter bacteriological grade Petri dishes in differentiating medium containing high-glucose Dulbecco's modified Eagle's medium (Gibco, Waltham, MA, USA), 15% fetal bovine serum, 2 mM GlutaMax, 1% non-essential amino acids, and 100 μM β-mercaptoethanol. embryoid bodies were replaced with fresh differentiation medium every other day.

### Statistical analysis

The Student’s *t*-test was used to analyze significant differences. *P*<0.05 was considered significant. The data analyses were performed using Prism GraphPad software.

### Accession codes

CGH data are deposited at the Gene Expression Omnibus under accession number GSE67563.

## Figures and Tables

**Table 1:** Developmental efficiencies of ICAHCI embryos

*Donor AG-haESCs*	*Passage number*	*Embryo stage*	*Number of transferred embryos*	*No. of normal pups (% of transferred embryos)*	*No. of pups surviving to adulthood (% of transferred embryos)*	*No. of Growth-retarded pups (% of transferred embryos)*
OG-3^a^	p22	Two-cell embryo	102	0	0	3 (2.9)
*H19*^*Δ1*^	>p56	Two-cell embryo	80	3 (3.8)	2 (2.5)	0
*H19*^*Δ1*^*-neo*^*Δ1*^	—	Two-cell embryo	50	1 (2)	ND	0
Round spermatids	—	Two-cell embryo	61	5 (8.2)	5 (8.2)	0

Abbreviations: AG-haESCs, androgenetic haploid embryonic stem cells; ICAHCI, intracytoplasmic AG-haESCs injection; ND, not determined.

a The data were from Yang *et al*.^7^
