# Supplementary material for: Rapidly generating knockout mice from H19-Igf2 engineered androgenetic haploid embryonic stem cells
Source: Cell Discov. 2015 Nov 3;1:15031–. doi: 10.1038/celldisc.2015.31 (PMC4860787; doi:10.1038/celldisc.2015.31)
Supplement: Supplementary information [file celldisc201531-s1.doc]

Supplementary Materials for

**Rapidly generating knockout mice from *H19-Igf2* engineered haploid ES cells**

Meili Zhang^1, 2, 5^, Yufang Liu^1, 2, 5^, Guang Liu^1, 2, 5^, Xin Li^3, 5^, Yuyan Jia^1, 2^, Lihong Sun^4^, Liu Wang^3^, Qi Zhou^3^ & Yue Huang^1, 2^

**This file includes:**

Supplementary Tables 1, 2

**Supplementary Table 1. Segmentation Table of the CGH analysis of *H19^Δ^* AG-haESCs**

| **Region** | **Log2_Ratio** |
| --- | --- |
| ***H19^Δ1^* vs AGH-OG-3** | |
| chr2:146813807-146832222 | -1.821030 |
| chr16:59445252-59999697 | 0.891244 |
| chr17:71112826-71404932 | 0.891020 |
| chr3:136446249-136571422 | 0.818407 |
| ***H19^Δ2^* vs AGH-OG-3** | |
| chr9:100658274-100701368 | -3.452282 |
| chr3:136446249-136571422 | -3.190809 |
| chr5:81801562-81883668 | 0.651543 |
| chr2:112290454-113249742 | 0.645194 |
| chr14:93593306-93603958 | 0.575491 |
| chr6:3131098-3571947 | 0.558617 |
| chr12:3102541-49267874 | 0.549672 |

**Supplementary Table 2. Primer sequences**

| **Category** | **Name** | **Sequence (5’-3’)** |
| --- | --- | --- |
| sgRNA | sgRNA-1 | CACCGACTGCTTTGCCCGTTCTTC |
|  |  | AAACGAAGAACGGGCAAAGCAGTC |
|  | sgRNA-2 | CACCGAGGCACCATTTCATATTCAT |
|  |  | AAACATGAATATGAAATGGTGCCTC |
|  | sgRNA-3 | CACCGCTTCAATATAATGCGACTCA |
|  |  | AAACTGAGTCGCATTATATTGAAGC |
|  | sgRNA-4 | CACCGACCACTGCAGGCATCGACGT |
|  |  | AAACACGTCGATGCCTGCAGTGGTC |
|  | sgRNA-5 | CACCGAGAAGTGGCCCCGGTGCATA |
|  |  | AAACTATGCACCGGGGCCACTTCTC |
|  | sgRNA-6 | CACCGAACTGGGTACTAGTCCAAT |
|  |  | AAACATTGGACTAGTACCCAGTTC |
|  | sgRNA-7 | CACCGTCCCTCTCACATCCTGCTC |
|  |  | AAACGAGCAGGATGTGAGAGGGAC |
|  | sgRNA-8 | CACCGCGAGGCTGTTTCCCACACTG |
|  |  | AAACCAGTGTGGGAAACAGCCTCGC |
| Surveyor assay | S1 | CCCTGTCCTCCCTGGATATT |
|  | S2 | CAACTCCCGAGGCATAATGT |
|  | S3 | CCAGCCTCTCTGTCTTGAGG |
|  | S4 | GGGAAAGAAATGAGGGAAGG |
|  | S5 | CCTATTCCCCATTCCATCCT |
|  | S6 | AAACCTCTTTGGCAATGCTG |
|  | S7 | TGAGGAAAGCAGGTCAGGTT |
|  | S8 | GCCCCTTTTGCTCTATGTCA |
|  | S9 | CTCCTGCAGGCTGGTCTTAC |
|  | S10 | CCTTGGTCAGCACCACTTTT |
|  | S11 | ACTGTGAGTCAGGGGTCTGG |
|  | S12 | ACCAGACTGGAGCCGAGTTA |
| Off-target | NR_046192-F | CGGAGATGAAAGTGCTGGGACA |
|  | NR_046192-R | TTTTGCTGTTCTTACCAGGGTCCT |
|  | NM_027045-F | CCACAGTGGAGGCTTGTTTGC |
|  | NM_027045-R | CCTTCTTCATTATGCCTCCCTAC |
|  | NM_001037927-F | GCTCAAAGCTAATGTACTGTTGGGTC |
|  | NM_001037927-R | GTGGAGATACAGCAAACAGTGGCT |
|  | NM_029427-F | GGGCAGAAGGTCACAGAACAGTC |
|  | NM_029427-R | TTCAGTGCCAGGGAAGGTGC |
|  | NM_013470-F | GAGGGCTCAAAACACAAACACTG |
|  | NM_013470-R | ACCTGATGTTCCGTGGTTGTAGA |
|  | NM_181422-F | AGTAAAGAACTCAGCGGACCCAAC |
|  | NM_181422-R | TTTGCTGGTCCAGTGGGAGTTT |
|  | NM_001081057-F | CCTGAAGCGTTCCATGTCCTAG |
|  | NM_001081057-R | AGGACCAGGCTGCTCATCAAG |
|  | NM_134142-F | GCATAGCAACGAGGATCTGGGT |
|  | NM_134142-R | CGCTTCCGTTCCAACTCCAAT |
| *H19* targeting vector | Left arm-F | GCACTTTCGGCCGCTCTCTAATGAGGACTGGTAGGAAC |
|  | Left arm-R | TCCTTCTTAATTAAGAGATGACTGACTGCCTGTCTTCC |
|  | Right arm-F | TGAATATCTCGAGCATAACTTCGTATAGCATACATTATACGAAGTTATATTAAAAAATGCCAGAGTGCCATACGG |
|  | Right arm-R | CCTCTTTGTCGACCTCAAAGTGAAGGGTTTTACCAATC |
| *H19^Δ^* | P1 | CTCTGTGGCACTATATGCCATGGTC |
|  | P2 | CCTACCCGGTAGAATTAATTCGATA |
|  | P3 | GAAATTGCATCGCATTGTCTGAGTA |
|  | P4 | GAGTGCCTTGTCTGTAAAGAAGCAT |
| *H19^Δ1^-neo^Δ^* | P5 | TCTTTATGTGGCTGTGGTCTACGC |
|  | P6 | CACATTGTTTGATGGCACTTGATT |
| RT-qPCR | *H19*-F | CATGTCTGGGCCTTTGAA |
|  | *H19*-R | TTGGCTCCAGGATGATGT |
|  | *Igf2*-F | CTAAGACTTGGATCCCAGAACC |
|  | *Igf2*-R | GTTCTTCTCCTTGGGTTCTTTC |
|  | *Snrpn*-F | TGCTACGTGGGGAGAACTTG |
|  | *Snrpn*-R | CCTGGGGAATAGGTACACCTG |
|  | *Grb10*-F | TCCAAGTGGAGAGTACCATGC |
|  | *Grb10*-R | TACGGATCTGCTCATCTTCG |
|  | *Gapdh*-F | CACTCTTCCACCTTCGATGC |
|  | *Gapdh*-R | CTCTTGCTCAGTGTCCTTGC |
| DMR  Bisulphite sequencing | *Snrpn*-BS-OF | TATGTAATATGATATAGTTTAGAAATTAG |
|  | *Snrpn*-BS-OR | AATAAACCCAAATCTAAAATATTTTAATC |
|  | *Snrpn*-BS-IF | AATTTGTGTGATGTTTGTAATTATTTGG |
|  | Snrpn-BS-IR | ATAAAATACACTTTCACTACTAAAATCC |
|  | *Gtl2*-BS-OF | TTAAGGTATTTTTTATTGATAAAATAATGTAGTTT |
|  | *Gtl2*-BS-OR | CCTACTCTATAATACCCTATATAATTATACCATAA |
|  | *Gtl2*-BS-IF | TTAGGAGTTAAGGAAAAGAAAGAAATAGTATAGT |
|  | *Gtl2*-BS-IR | TATACACAAAAATATATCTATATAACACCATACAA |
